# Triacetin and a Mushroom Blend Restore Butyrate Production by IBS Microbiomes Ex Vivo, Thus Promoting Barrier Integrity

**DOI:** 10.3390/ijms26199388

**Published:** 2025-09-25

**Authors:** Pieter Van den Abbeele, Jonas Poppe, Aurélien Baudot, Lam Dai Vu

**Affiliations:** Cryptobiotix SA, Technologiepark-Zwijnaarde 82, 9052 Ghent, Belgium; jonas.poppe@cryptobiotix.com (J.P.); aurelien.baudot@cryptobiotix.com (A.B.); lamdai.vu@cryptobiotix.com (L.D.V.)

**Keywords:** triacetin, mushroom, prebiotic, butyrate, barrier integrity, SIFR, SCFA, IBS

## Abstract

Irritable Bowel Syndrome (IBS) is a common gastrointestinal disorder, characterized by abdominal pain, altered bowel habits (diarrhea and/or constipation) and a dysbiosis of the gut microbiome. This dysbiosis is difficult to restore via fiber supplementation, which typically promotes gas production, potentially worsening IBS symptoms. We therefore studied how two novel products, triacetin (TA; REBiome™) and a mushroom blend (MB; Hōlistiq™), modulate the microbiome of IBS subjects (*n* = 8) using the ex vivo SIFR^®^ (Systemic Intestinal Fermentation Research) technology combined with a co-culture of epithelial/immune (Caco-2/THP-1) cells. First, the IBS microbiomes revealed large interpersonal variability and an IBS-associated dysbiosis. TA increased the beneficial metabolites acetate and butyrate (~*Anaerobutyricum soehngenii, Mediterraneibacter*_A *butyricigenes*, *Faecalibacterium prausnitzii*). Moreover, MB stimulated a wide range of gut microbes and additionally promoted propionate. Despite more strongly increasing total short-chain fatty acid (SCFA) levels, TA induced significantly less gas production than MB. Mechanistically, acetate with TA was derived from hydrolysis, a process that indeed does not induce gas production. Notably, both TA and MB enhanced gut barrier integrity (transepithelial electrical TEER), which is related to lower symptom severity in IBS patients. Overall, our findings highlight the product-specific microbiome modulation and potential of MB, TA or combinations thereof as dietary interventions for managing IBS symptom severity.

## 1. Introduction

The gut microbiome plays a crucial role in human health [1,2], among others, by producing short-chain fatty acids (SCFAs) from glycans [3,4]. The main SCFAs are acetate, propionate and butyrate, which each have specific benefits [5]. Disruptions in the microbiome are associated with diseases like Irritable Bowel Syndrome (IBS) [6], one of the most common gastrointestinal disorders [7,8]. Key symptoms of IBS are abdominal pain or discomfort and altered bowel habits, i.e., diarrhea (IBS-D), constipation (IBS-C) or a mix thereof (IBS-M) [9]. The potential of microbiome-targeted strategies was confirmed by El-Salhy et al. (2020), who demonstrated that fecal microbiome transplantation from a healthy donor to IBS subjects improved symptom scores [10]. As IBS-associated microbial dysbiosis (especially IBS-D) is linked to a depletion of butyrate-producing microbes [6], restoring butyrate production seems a promising strategy. Mechanistically, butyrate could restore gut barrier integrity [11], which is relevant in IBS given that IBS patients suffer from an impaired barrier integrity [12] that is linked to symptom severity [13,14].

The stimulation of SCFA such as butyrate is usually achieved by consumption of dietary fibers [3,15]. Pathways involved in the production of SCFA, particularly butyrate, typically involve the production of gases such as H_2_ and CO_2_ [5,16]. This is problematic for IBS patients that are often on a low FODMAP diet, i.e., a diet low in fermentable oligosaccharides, disaccharides, monosaccharides and polyols [17], in an attempt to minimize colonic gas production and associated discomfort. An additional issue is that the outcome of colonic fermentation of fibers can be prone to large interpersonal differences [18], which cause unpredictable treatment outcomes. There is thus a need to develop products that promote SCFA production, in particular butyrate, without causing intestinal discomfort.

While clinical studies are essential to demonstrate health benefits, they are ill suited to gain mechanistic insights into microbiome modulation. First, metabolites such as SCFA are readily absorbed [19], rendering fecal samples non-informative on metabolite production in the colon. In addition, fecal microbiome composition is confounded by multiple factors that all together cause great heterogeneity in microbiota profiles, thus obscuring insights into true associations with interventions or diseases [20,21]. The ex vivo SIFR^®^ technology (Cryptobiotix, Ghent, Belgium) (Systemic Intestinal Fermentation Research), which employs high-throughput, reactor-based simulations of the gut microbiome, was recently introduced to overcome such limitations. The technology was validated to provide predictive insights for clinical studies, not only in terms of microbiome modulation [22] but also for effects on barrier integrity and immune modulation [23]. Such clinical predictivity stems from the ability of the technology to cultivate microbiomes in a way that they still highly resemble the original in vivo microbiome, a distinctive feature compared to legacy in vitro gut models [22].

Our aim was to evaluate whether two novel products with unknown microbiome-modulating potential, triacetin (TA; triacyl glyceride of acetate; REBiome™, Compound Solutions, Carlsbad, CA, USA) and a mushroom blend (MB; a new prebiotic β-glucan source [24]; Hōlistiq™, Compound Solutions, Carlsbad, CA, USA), impact the IBS microbiome, using the ex vivo SIFR^®^ technology (Cryptobiotix, Ghent, Belgium). Notably, TA and MB restored butyrate production, each via different pathways. A unique effect of TA was that it only induced low gas production, while MB uniquely promoted propionate production and microbial diversity. A key finding was that both TA and MB enhanced gut barrier integrity, suggesting that both products are promising therapeutic agents for improving IBS-related disease symptoms.

## 2. Results

### 2.1. The IBS Microbiome Displayed Large Interpersonal Differences and an IBS-Associated Dysbiosis

**Figure 1:**
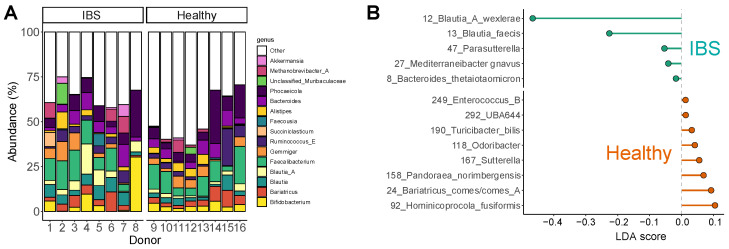
**The fecal microbiota of IBS subjects displayed marked interpersonal differences and relevant differences compared to a healthy cohort**. (**A**) Abundances (%) of the most abundant genera for each IBS subject (subjects 1–8) and healthy adult (subjects 9–16). (**B**) Linear discriminant analysis effect size (LEfSe) showing the taxa that explained differences between both cohorts (*p* < 0.05).

The fecal microbiomes of the IBS subjects, used to assess the microbiome-modulating potential of TA and MB, displayed large interpersonal differences (Figure 1A). First, *Bifidobacteriaceae* ranged from virtually absent (subjects 2, 6, 7), over 2–3% (subjects 3, 5) and 5–10% (subjects 1, 4) up to 30% of the microbiota for subject 8, whose microbiota were in contrast depleted from *Ruminococcaceae* (e.g., *Faecalibacterium*, *Gemmiger*). Moreover, *Bacteroidaceae* genera (*Phocaeicola* and/or *Bacteroides*) were highly abundant for subjects 3, 4, 7 and 8. Covering such large interpersonal differences is essential to obtain representative insights into the microbiome-modulating potential of interventions.

The relevance of the IBS cohort was further confirmed by the observation of an IBS-specific phenotype compared to healthy adults (Figure 1B). Indeed, the IBS microbiota were enriched with OTUs related to *Blautia*_A *wexlerae*, *Blautia faecis* and *Mediterraneibacter gnavus* (formerly *Ruminococcus gnavus*), at the expense of Bacillota (formerly Firmicutes) OTUs including *Bariatricus comes* (formerly *Coprococcus comes*). Notably, *Bifidobacterium* species colonized the microbiota of healthy subjects more consistently compared to IBS subjects, with abundances ranging from 1.6 to 5.6% (Figure 1A).

### 2.2. Encapsulation Enhances Colonic Delivery of TA

To apply a relevant TA dose in the colon, the stability of TA along the upper gastrointestinal tract was first assessed. When dosed as such, a cumulative acetate release of 33.8% (±0.3%) was noted along the upper gastrointestinal tract, implying that 66.2% of TA escaped digestion. Upon encapsulation, the cumulative acetate release at the end of the small intestinal phase was 10.8% (±0.9%). This demonstrates that encapsulation improves colonic delivery of TA to 89.2%. The subsequent study focusing on how TA impacts the colonic microbiome simulated this scenario in which TA is encapsulated (=89.2% of the oral TA dose was administered at the start of the colonic phase).

### 2.3. TA and MB Boosted SCFA, Which Was Accompagnied by Only Low Gas Production for TA

**Figure 2:**
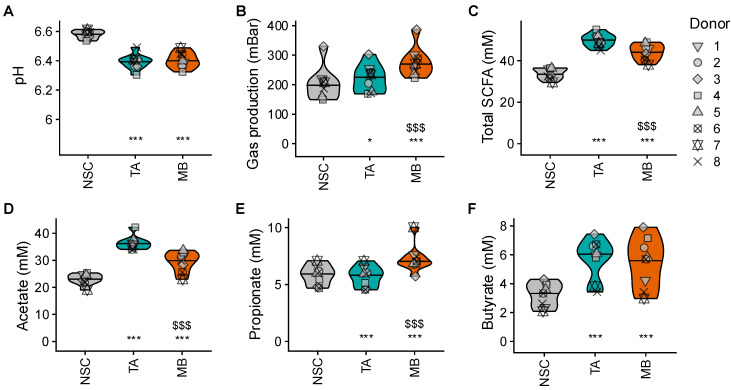
**TA and MB boosted SCFA levels in a product-specific manner with remarkably low gas production for TA.** The impact on (**A**) pH, (**B**) gas production (mbar), (**C**) total SCFA (mM), (**D**) acetate (mM), (**E**) propionate (mM) and (**F**) butyrate (mM). Statistical differences between TA or MB and the NSC are indicated with * (0.01 < p_adjusted_ < 0.05), ** (0.001 < p_adjusted_ < 0.01) or *** (p_adjusted_ < 0.001), while differences between TA and MB are indicated with $/$$/$$$.

TA and MB significantly lowered colonic pH, increasing gas production and total SCFA levels (Figure 2A–C). Notable product-specific effects included the following: while TA more specifically increased acetate and butyrate, MB significantly increased all three main SCFAs (also propionate) (Figure 2D–F). Moreover, while TA increased acetate and total SCFA significantly more strongly than MB, gas production was significantly lower with TA. This remarkably low gas production with TA was further stressed when calculating the ratio of gas production per mole of SCFA being generated: unlike MB, TA markedly and significantly decreased this ratio (Figure S1).

### 2.4. TA and MB Each Enhanced the Growth of Specific SCFA-Producing Gut Microbes

The product-specific effects on SCFA levels were confirmed by product-specific effects on microbial composition, further corroborating how TA and MB each trigger specific microbial pathways in the colon.

**Figure 3:**
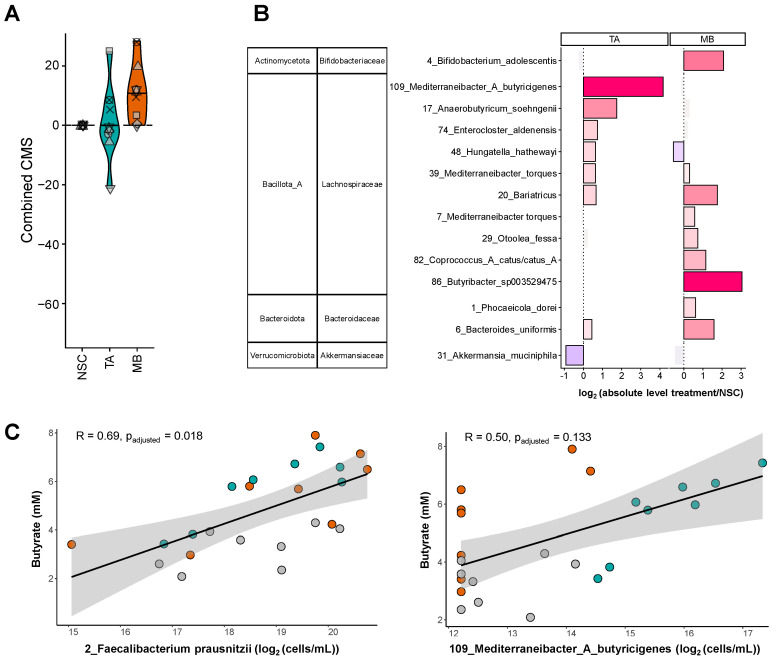
**TA and MB each enhanced the growth of specific SCFA-producing gut microbes**. The impact on (**A**) microbial diversity (community modulation score (CMS)), and (**B**) OTUs that were significantly affected (p_adjusted_ < 0.05), expressed as log_2_ transformation of the ratio of abundance treatment/abundance NSC, averaged across all 8 IBS subjects. Hence, values > 0 reflect a stimulation by TA/MB, while values < 0 indicate an inhibition by TA/MB. Bars highlighted by a black outline indicate a statistically significant increase/decrease. Color intensity is proportional to the extent of the effect (increase = red; decrease = pink). (**C**) Individual correlations between butyrate (mM) and specific OTUs based on Spearman’s rank correlation coefficient. The color of the circles illustrates the study arm of the corresponding data point (NSC = grey, TA = green, MB = red).

TA specifically increased a consortium of *Lachnospiraceae* members, with particularly strong effects on OTUs related to *Anaerobutyricum soehngenii* and *Mediterraneibacter*_A *butyricigenes* (Figure 3B). MB stimulated a broader range of OTUs related to species belonging not only to the *Lachnospiraceae* (e.g., *Bariatricus, Coprococcus* and *Butyribacter* species) but also to the *Bifidobacteriaceae* (e.g., *Bifidobacterium adolescentis*) and *Bacteroidaceae* families (e.g., *Phocaeicola dorei* and *Bacteroides uniformis*). The involvement of a larger number of taxa in the fermentation of MB was in line with the finding that MB specifically increased microbial diversity as quantified by the community modulation score (CMS) (Figure 3A).

Finally, strong correlations between butyrate levels and two specific species were noted, suggesting the involvement of these species, *Mediterraneibacter*_A *butyricigenes* (upon TA treatment) and *Faecalibacterium prausnitzii* (upon TA and MB treatment), in the production of butyrate (Figure 3C). For *F. prausnitzii,* this was not captured by the statistical analysis of treatment effects due to the large interpersonal differences in baseline levels of *F. prausnitzii* among IBS subjects. Nevertheless, its strong correlation with butyrate confirmed that *F. prausnitzii* is a key driver of butyrate production during microbial fermentation of TA and MB in the colon.

### 2.5. TA and MB Promoted Gut Barrier Integrity Under Non-Stressed and Stressed Conditions

**Figure 4:**
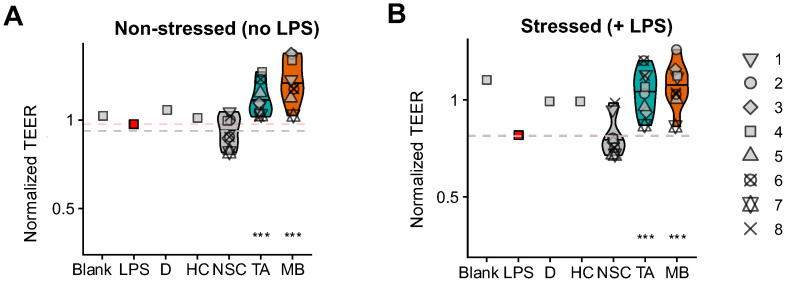
**TA and MB promoted epithelial gut barrier integrity, both under non-stressed and stressed conditions**. The host–microbiome interaction assay assessed barrier integrity (TEER, normalized to 0 h) under (**A**) non-stressed (24 h) and (**B**) stressed conditions (30 h = additional 6 h LPS treatment). References included cell medium (blank), LPS treatment (24–30 h; LPS = red square) and its co-supplementation of dexamethasone (D) or hydrocortisone (HC). The control to evaluate potential significance of treatment effects (=NSC) involved a study arm in which the IBS microbiota were cultured in the ex vivo SIFR^®^ technology in absence of any treatment. Both references, i.e., the blank and NSC (average across subjects) are highlighted with a dotted line. Statistical differences between TA or MB and the NSC are indicated with *** (p_adjusted_ < 0.001).

The stimulation of beneficial SCFA by specific gut microbes translated into strong and significant increases in gut barrier integrity (TEER) upon treatment with TA and MB, both under non-stressed and stressed conditions (Figure 4A,B). MB tended to increase barrier integrity more strongly, especially under non-stressed conditions. Further, both TA and MB tended to lower the production of the pro-inflammatory cytokine TNF-α while exerting rather neutral effects on IL-6, IL-10, IL-8 and IL-1β (Figure S2).

## 3. Discussion

The aim of this study was to decipher the microbiome-modulating potential of triacetin (TA, REBiome™) and a mushroom blend (MB, Hōlistiq™) when dosing the microbiome of IBS subjects, using the ex vivo SIFR^®^ technology. The use of representative IBS microbiota ensured representative findings on the microbiome-modulating potential of both products: the relevance of the IBS cohort followed from the large interpersonal differences that were covered along with the observation of an IBS-specific phenotype. In line with previous studies, the IBS microbiota were particularly enriched with OTUs related to *Blautia* species [25,26] and *Mediterraneibacter gnavus* [27,28], a species that was shown to have a pathogenic role in diarrhea-predominant IBS [29]. In addition, a subject-specific depletion of *Ruminococcaceae* was noted [6]. A key finding of the current study is that TA and MB strongly promoted gut barrier integrity, the hallmark of gut health in general [30], yet particularly relevant in IBS [12], where enhanced barrier integrity has been linked to lower symptom severity [13,14]. Notably, TA and MB exerted this beneficial effect through product-specific modulation of the gut microbiome.

Both TA and MB enhanced the SCFA butyrate, which has been shown to promote gut barrier integrity [11]. While the impact on butyrate and barrier integrity was similar for TA and MB, the underlying processes differed. TA specifically increased the butyrate producers *Mediterraneibacter*_A *butyricigenes* [31] and *Anaerobutyricum soehngenii* [32], while MB rather increased *Bariatricus, Coprococcus* [33] and *Butyribacter* species [34]. Moreover, both MB and TA stimulated *Faecalibacterium prausnitzii,* a species shown to not only exert beneficial effects via butyrate but additionally via the production of, for instance, anti-inflammatory peptides [35]. Another example of how these butyrate-producing gut microbes could contribute to health benefits is that *Anaerobutyricum soehngenii* is linked with metabolic health, particularly in obese and insulin-resistant individuals [36]. This aligns with observations that TA decreased de novo lipogenesis and in doing so lowered weight gain in an animal model of diet-induced obesity [37]. To further strengthen the link between microbiome changes and beneficial effects in diet-induced obesity, future studies could focus on the impact of TA in obese test subjects, given that the present study focused on subjects with a BMI between 20 and 25. Taken together, these findings suggest that TA and MB can promote butyrogenic members of the gut microbiome, thus restoring the IBS-associated dysbiosis that is characterized by a depletion in such microbes [6]. Moreover, the stimulation of butyrate by TA and MB is likely a key mechanism by which MB and TA promote barrier integrity that contributes to lower symptom severity in IBS.

A notable observation for TA was that its strong increase in SCFA levels was accompanied by only low gas production. This is of interest as previous research in the field has focused on identifying treatments that attenuate gas production upon fiber consumption, aiming at providing benefits via SCFA while minimizing discomfort for IBS patients [38,39]. Mechanistically, the low gas production with TA may be explained by the hydrolysis of TA, which directly releases glycerol and (up to) three molecules of acetate [40] without yielding gases that are instead typically produced when gut microbes ferment fibers into SCFA. While subsequent acetate-into-butyrate cross-feeding interactions noted for TA did yield gases likely including CO_2_ and H_2_ [5,16], overall gas production with TA was thus attenuated. Another surprising finding was that the experimentally observed increases in acetate and butyrate exceeded the theoretically dosed amounts of acetate (as part of TA), i.e., 123.2% of carbon was recovered (91.5% as acetate, 31.7% as butyrate). This suggests that additional synergistic interactions take place, potentially including the fact that TA lowers colonic pH, thus favoring the growth of butyrate producers (that indeed thrive better at lower pH) over other gut microbes [41]. In addition, some of the carbon atoms for the additional 23.2% could be derived from fermentation of the glycerol backbone. Indeed, glycerol can be fermented into lactate [42], which is a precursor of butyrate [32]. Overall, this suggests that TA is an effective product for delivering SCFA to the body without excessive gas production, as demonstrated under the tested ex vivo conditions. A notable product-specific effect of MB was that it also increased propionate production, mediated via *B. uniformis* and *P. dorei*. *Bacteroides* and *Phocaeicola* species indeed commonly utilize the succinate pathway to convert succinate into propionate [4]. Previous studies have shown that beneficial effects of propionate include the fact that it promotes satiety, lowers blood cholesterol and improves insulin sensitivity [5]. Further, MB uniquely promoted *Bifidobacteriaceae,* another family containing distinct health-related members of the gut microbiome [43,44]. The involvement of these taxa in the fermentation of MB aligns with earlier studies with mushrooms and likely relates to fiber constituents of mushrooms such as chitin, β-glucan and hemicellulose [24]. Compared to TA, the fermentation of MB involved a larger spectrum of gut microbes, causing MB to increase microbial diversity, as assessed with the community modulation score (CMS; Figure 3A). Notably, traditional α-diversity indices were unable to capture this stimulatory effect of MB on a broader range of community members (Figure S3). Unlike the CMS, these traditional α-diversity indices are biased as they do not correct for the lower sequencing depth in communities with higher cell density [45]. As MB promotes cell density (Figure S4), diversity is systematically underestimated by traditional indices. Overall, the promotion of microbial diversity by MB might be beneficial to restore the lower diversity observed in the microbiome of IBS patients [6].

Finally, prior to the assessment of the impact of TA on the gut microbiome, we evaluated its digestion along the simulated upper gastro-intestinal tract. This revealed how encapsulation is a valid strategy to increase colonic delivery of TA as it lowered digestion from 33.8% down to 10.8%. Even without encapsulation, digestion of TA was rather limited (33.8%), which is in line with previous findings showing how triacetin is less impacted by lipases compared to for instance tributyrin [46]. These findings were not in line with a rat study during which triacetin was fully digested along the upper GIT [47]. This could relate to the fact that TA was not encapsulated while likely also relating to the vastly different host physiology between rodents and humans [48].

Overall, MB and TA exerted product-specific effects on the microbiome of IBS patients. Both products restored the butyrogenic capacity of the microbiome and promoted barrier integrity, highlighting their potential as dietary interventions for managing IBS symptom severity. Moreover, as many observations were product-specific, combinations of both products could be of interest to potentiate effects. Replacing some MB with TA could, for instance, lower gas production while further increasing SCFA levels. In turn, replacing some TA with MB could promote also propionate production and increase microbial diversity. As both products act on different butyrate producers that are not provided as part of the product but should be present in the background microbiota, combining both products could ensure that a butyrate producer is present in the microbiome of a given IBS patient, even when severely dysbiosed. This could limit interpersonal differences in response to interventions. It will be interesting to assess how both products, either as such or in combination, could improve IBS symptom scores in clinical studies.

## 4. Materials and Methods

### 4.1. Selection Criteria Test Subjects

To study the microbiome-modulating effect of TA and MB for IBS subjects ex vivo, fresh fecal samples of eight IBS subjects were collected. Subjects with both IBS-M (mixed type) and IBS-D (diarrhea type) were included, as defined by Rome IV criteria [9]. IBS-C patients (constipation type) were not considered as stool consistency and colonic transit time are among the strongest determinants of microbial composition [20], so that including slow-transit IBS-C patients could introduce a confounding factor.

They additionally complied with the following criteria: age 30–65, no antibiotic/probiotic intake in 3 months prior to study, no other gastro-intestinal disorders (cancer, ulcers, IBD) and 20 < body mass index < 25 (to avoid inclusion of overweight or obese subjects, which could also introduce a confounding factor [49]). No specific diet diaries were collected. This resulted in the enrolment of 8 specific IBS subjects with an average age of 42.8 (±6.8) years.

To understand whether the microbiomes of these IBS subjects displayed an IBS-associated dysbiosis, fecal microbiota of healthy human adults were sourced. These healthy adults complied with the same criteria as the IBS subjects (except that they did not suffer from IBS-M or IBS-D). The healthy subjects had an average age of 38.0 (±4.3) years.

### 4.2. Upper Gastrointestinal Tract Simulation

The simulated upper gastrointestinal tract passage of TA was performed as described before [50]. Briefly, TA, both as such and upon encapsulation, was exposed to an oral, gastric and small intestinal phase, during which digestive secretions were sequentially added. Samples were collected at the end of each phase and analyzed for SCFA content to assess the potential release of the SCFA acetate from TA. As acetate released along the upper gastrointestinal tract would be absorbed in vivo, it was important to accurately estimate colonic delivery of TA (intact TA fraction at the end of the upper gastrointestinal tract = 100%—measured free acetate level at end of upper gastrointestinal tract/theoretically dosed acetate as part of TA). In doing so, a biorelevant (non-digestible) fraction of the oral TA dose could be administered at the colonic incubations.

### 4.3. Ex Vivo Colonic Fermentations (SIFR^®^): Experimental Configuration, Timeline and Analysis

Colonic fermentations were performed as described before [22]. Briefly, bioreactors containing 5 mL of a blend of nutritional medium (M0028, Cryptobiotix, Ghent, Belgium), fecal IBS microbiota and a specific test product (TA or MB) were rendered anaerobic using a bioreactor management device (Cryptobiotix, Ghent, Belgium) and incubated under constant agitation (140 rpm) at 37 °C (MaxQ 6000, Thermo Scientific, Merelbeke, Belgium). Directly upon collection of fresh fecal samples, fecal slurries were prepared in anaerobic phosphate buffer (0.05 M). These slurries were inoculated at a final average cell density of 8.45 × 10^8^ cells/mL colonic suspension (Figure S5).

**Figure 5:**
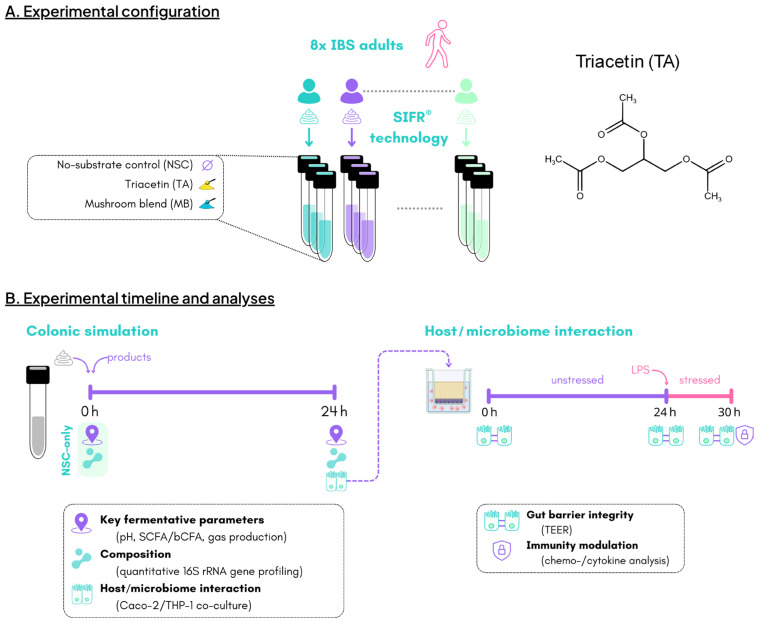
The impact of TA and MB on the gut microbiota of IBS subjects was assessed using the ex vivo SIFR^®^ technology (*n* = 8). (**A**) Experimental configuration, (**B**) timeline and analysis.

This study consisted of three arms that were each assessed for eight individual IBS subjects (*n* = 8). This included the unsupplemented control (no substrate control; NSC), triacetin (TA; REBiome™, Compounds solutions, Carlsbad, CA, USA) and a mushroom blend (MB; Hōlistiq™, Compounds solutions, Carlsbad, CA, USA) (Figure 5A). MB consisted of 72% (*w*/*w*) carbohydrates (of which 12.3% dietary fiber and 6.4% sugar), 13% protein and 6.4% fat. It contained the following minerals (mg/100g): Ca (67), Cu (0.32), Fe (4.9), Mg (123), Mn (3.6), P (293), K (300), Na (10) and Zn (2.3). In contrast to the products that were tested in *n* = 1 for each IBS subject, the NSC arm was run in *n* = 3 for each subject to confirm the high technical reproducibility of the SIFR^®^ technology.

TA was tested at a dose simulating the oral ingestion of 400 mg TA as part of a capsule. MB was tested at a daily dose of 1000 mg and consisted of 50% *Cordyceps militaris*, 30% *Ganoderma lucidum* (Reishi), 5% *Hericium erinaceus* (Lion’s mane), 5% *Lentinula edodes* (Shiitake), 5% *Pleurotus eryngii* (King trumpet) and 5% *Trametes versicolor* (Turkey tail).

After 24 h of incubation, gas pressure was measured in the headspace of reactors and liquid samples were collected for assessment of key fermentative parameters (pH, gas and SCFA production), microbial composition and host/microbiome interactions (Figure 5B). Treatment effects of TA and MB were established by comparing the study arms treated with TA/MB to the NSC study arm. Indeed, the latter (NSC) involved growing the IBS microbiomes under identical conditions, except without TA/MB supplementation.

### 4.4. Key Fermentative Parameters

SCFA (acetate, propionate and butyrate) were determined via gas chromatography with flame ionization detection, as previously described [51]. Briefly, samples were diluted, acidified and supplemented with sodium chloride and internal standard, and extracted with diethyl ether. Extracts were analyzed using a Trace 1300 chromatograph equipped with a Stabilwax-DA capillary GC column, a flame ionization detector and a split injector using nitrogen gas as the carrier and makeup gas (Thermo Fisher Scientific, Merelbeke, Belgium). Finally, pH was measured using an electrode (Hannah Instruments Edge HI2002, Temse, Belgium).

### 4.5. Microbial Composition

Microbial composition was analyzed via quantitative 16S rRNA gene profiling targeting the V3–V4 region. Briefly, cell densities per individual operational taxonomic unit (OTU) were estimated by multiplying relative abundances (%) obtained via 16S rRNA gene sequencing with total cell numbers of each sample (cells/mL; flow cytometry). First, DNA was extracted via the SPINeasy DNA Kit for Soil (MP Biomedicals, Eschwege, Germany). Subsequently, library preparation and sequencing were performed on an Illumina MiSeq platform with v3 chemistry (2 × 300 bp). Amplicons were generated via the primers 341F (50-CCT ACG GGN GGC WGC AG-30) and 785Rmod (50-GAC TAC HVG GGT ATC TAA KCC-30). Pre-processing and OTU picking were performed with Mothur v1.35.1 [52], including annotation of representative sequences with NCBI blast v2.10.0 [53]. Total cell numbers were quantified by diluting samples in PBS, staining cells with SYTO 16 and counting them via a NovoCyte flow cytometer (Agilent, Santa Clara, CA, USA).

### 4.6. Host/Microbiome Interaction Assay

To study the impact of colonic samples (upon TA/MB treatment) on host cells, a co-culture experiment with epithelial cells (human adenocarcinoma Caco-2 cell line, ATCC^®^ HTB-37™) and immune cells (human acute monocytic leukemia THP1 cell line, differentiated to activated macrophages by a PMA treatment, ATCC^®^ TIB-202™) was performed, as previously described [23]. Both cell lines were obtained from the American Type Culture Collection (ATCC, Manassas, VA, USA). Briefly, the gut wall was created by covering immune cells with an epithelial layer. The host–microbiome interaction assay had two phases: (i) 24 h treatment with colonic samples to evaluate gut barrier integrity under non-stressed conditions, and (ii) 6 h additional incubation with lipopolysaccharide (Ultrapure LPS from *E. coli* 0111:B4, category number = tlrl-3pelps, Invitrogen, Carlsbad, CA, USA) to evaluate effects under stressed conditions. Colonic samples were centrifuged and filter-sterilized before use in the assay (administered apically as 20% of the cell medium: MEM medium supplemented with 1× NEAA and 1 mM Sodium Pyruvate with 10% FBS, Gibco, Gibco, Carlsbad, CA, USA). Besides SIFR-derived colonic samples, extra controls were run to confirm the experiment was performed optimally. These controls consisted of replacing the cell medium at 24 h with cell medium containing no extra treatment (Blank), LPS and the anti-inflammatory compounds dexamethasone (D) or hydrocortisone (HC). The latter are two positive controls that avoid LPS-induced inflammation and disruption of barrier integrity. Transepithelial electrical resistance (TEER) measured gut barrier integrity at 0 h, 24 h and 30 h. Immune effects at 30 h were studied via cytokine/chemokine production on the basolateral side using a Multiplex Luminex^®^ Assay kit on the MAGPix^®^ analyzer (Luminex Corporation, Austin, TX, USA) (IL-6, IL-10, TNF-α and IL-1β) or ELISA (IL-8). The lowest standards included in the standard curve had a concentration of 1.37, 1.39, 3.06, 7.57 and 31.30 pg/mL for IL-6, IL-10, TNF-α, IL-1β and IL-8, respectively, while the highest standards involved testing 1000, 1010, 2230, 5520 and 2000 pg/mL. To ensure quantification within this range, SIFR-derived samples were diluted 1/10 for all cytokines except for IL-8, for which a dilution of 1/500 was applied. For quality control purpose, all individual samples were run in *n* = 2 and analyzed for TEER, with the first replicate being analyzed for cytokine/chemokine production.

### 4.7. Data Analysis and Statistics

Data analysis was performed using R (version 4.4.0; www.r-project.org; accessed on 26 May 2025). Statistical analysis was conducted using a linear mixed-effects model, which accounted for both fixed effects (treatment) and random effects (interpersonal variation). The Benjamini–Hochberg correction was applied to control the false discovery rate across parameters. Effects of TA or MB compared to the reference (NSC) were assessed via a post hoc pairwise comparison, again with Benjamini–Hochberg correction of *p*-values. Effects were significant at p_adjusted_ < 0.05. In the violin plots, statistical differences between TA or MB and the NSC are indicated with * (0.01 < p_adjusted_ < 0.05), ** (0.001 < p_adjusted_ < 0.01) or *** (p_adjusted_ < 0.001), while differences between TA and MB are indicated with $/$$/$$$. Finally, pairwise correlation analysis using Spearman’s rank correlation coefficient was performed between SCFA and absolute levels of different OTUs.

## Data Availability

The raw data supporting the conclusions of this article will be made available by the authors on reques.

## Supplementary Materials

The following supporting information can be downloaded at https://www.mdpi.com/article/10.3390/ijms26199388/s1.

## Abbreviations

The following abbreviations are used in this manuscript:

BSS	Bristol stool score
CMS	Community modulation score
IBS	Irritable Bowel Syndrome
MB	Mushroom blend
NSC	No-substrate control
OTU	Operational taxonomic unit
SCFA	Short-chain fatty acid
SIFR^®^	Systemic Intestinal Fermentation Research
TA	Triacetin
TEER	Transepithelial electrical resistance
