# Supplementary material for: Triacetin and a Mushroom Blend Restore Butyrate Production by IBS Microbiomes Ex Vivo, Thus Promoting Barrier Integrity
Source: Int J Mol Sci. 2025 Sep 25;26(19):9388. doi: 10.3390/ijms26199388 (PMC12524982; doi:10.3390/ijms26199388)
Supplement: Supplementary file 1 [file ijms-26-09388-s001.zip › ijms-3818749-supplementary.pdf]

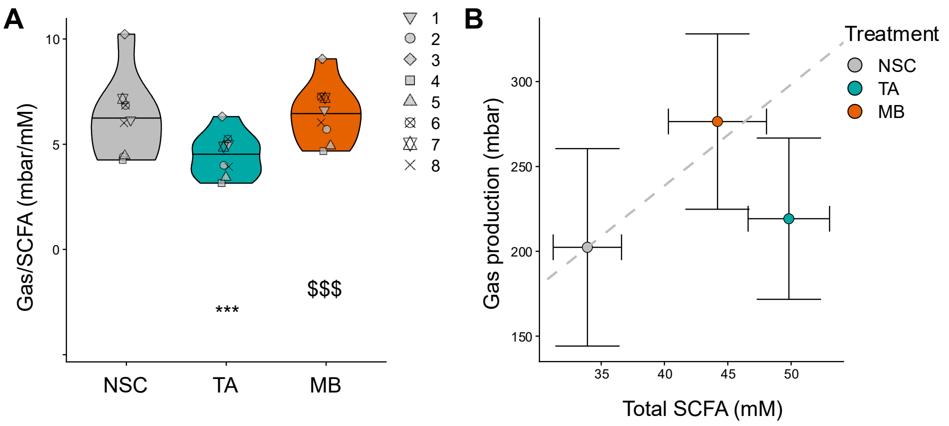

Figure S1. The strong increase in SCFA levels with TA was accompanied by only mild increases in gas production. (**A**) Ratio of gas production per mole of SCFA being produced (mbar/mM). (**B**) Absolute gas production (mbar) in function of total SCFA production (mM).

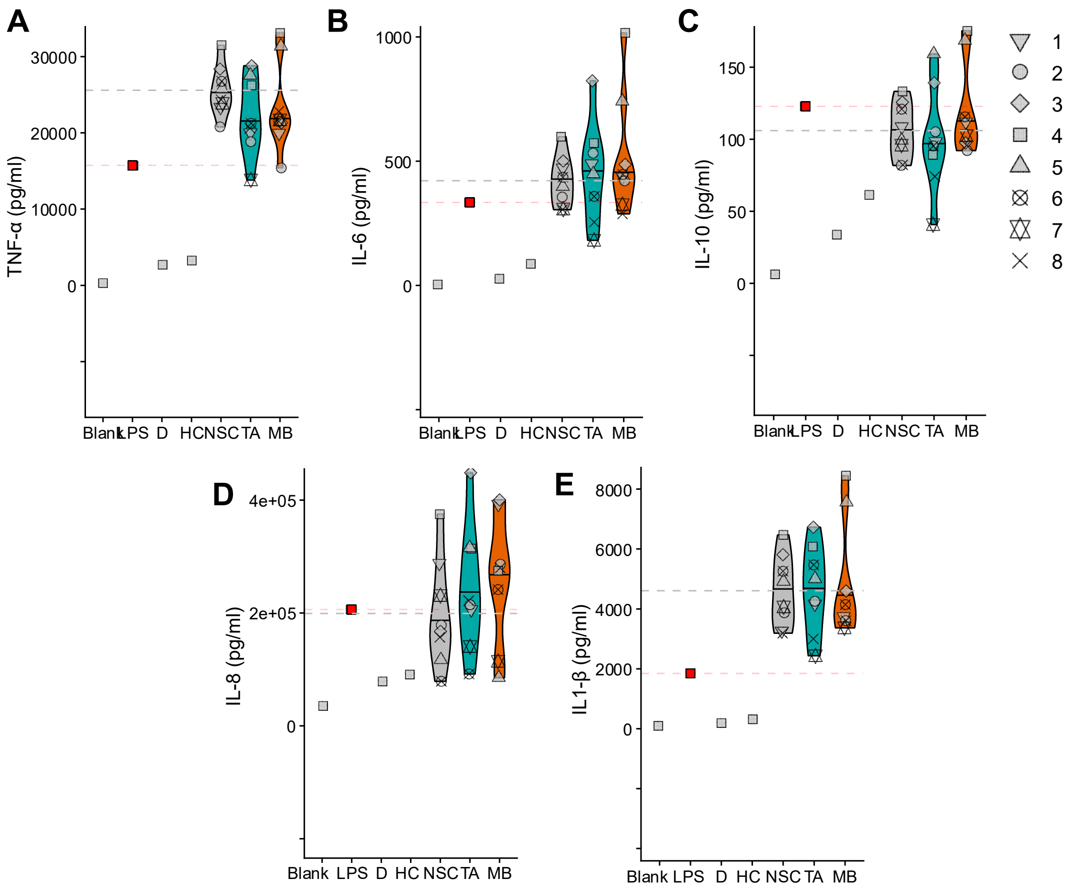

Figure S2. TA and MB tended to lower TNF-α, while exerting neutral effects on IL-6 and IL-10. The host–microbiome interaction assay assessed (**A**) TNF-α, (**B**) IL-6, (**C**) IL-10, (**D**) IL-8 and (**E**) IL-1β at 30h, i.e. upon 24h of interaction between test products and the human cells, followed by LPS treatment for an additional 6h period. References included cell medium alone (blank), LPS treatment alone (between 24-30h) and co-supplementation of dexamethasone (**D**) or hydrocortisone (HC). The control to evaluate potential significance of treatment effects (= NSC) involved a study arm in which the IBS microbiota was cultured over 24h in the *ex vivo* SIFR® technology in absence of any treatment.

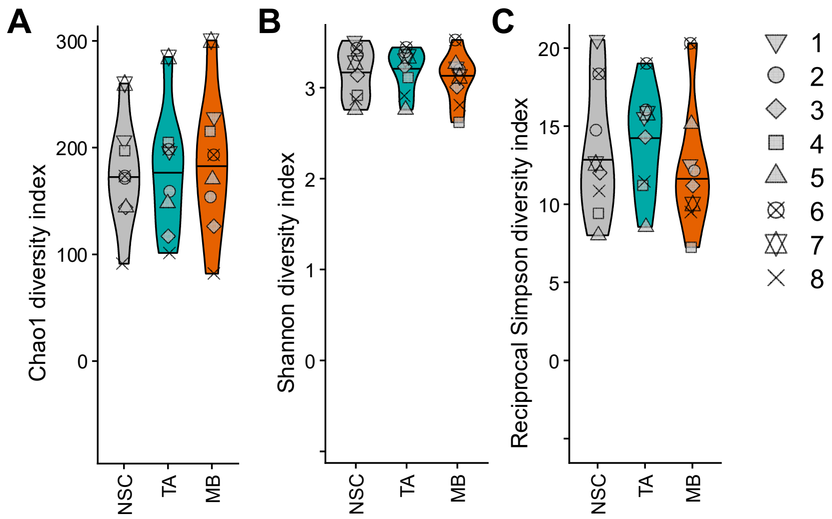

Figure S3. TA and MB did not impact traditional microbial diversity indices. The impact on (**A**) the Chao1 diversity index, (**B**) Shannon diversity index and (**C**) reciprocal Simpson diversity index.

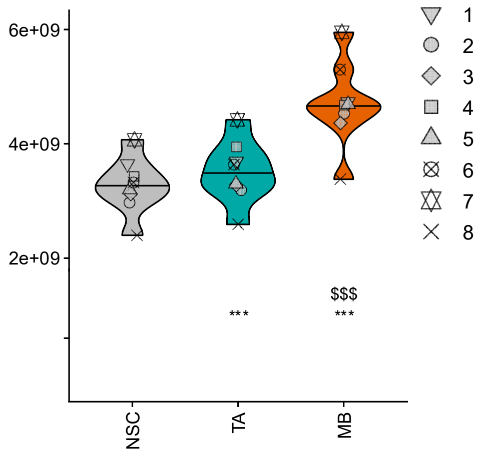

Figure S4. TA and especially MB promoted microbial density (cells/mL) upon 24h of incubation.

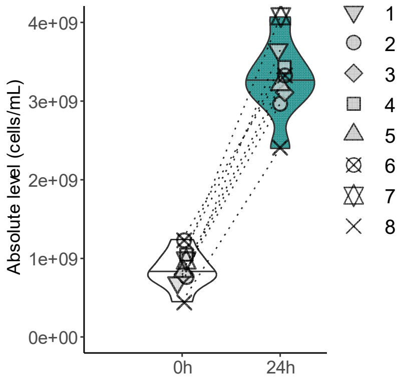

Figure S5. Cell density (cells/mL) in the untreated parallel control (NSC) upon inoculation (0h) and upon 24h of incubation.
